# PIMS sequencing extension: a laboratory information management system for DNA sequencing facilities

**DOI:** 10.1186/1756-0500-4-48

**Published:** 2011-03-07

**Authors:** Peter V Troshin, Vincent LG Postis, Denise Ashworth, Stephen A Baldwin, Michael J McPherson, Geoffrey J Barton

**Affiliations:** 1 College of Life Sciences, University of Dundee, Dundee, DD1 4HN, UK; 2 Institute of Membrane and System Biology, University of Leeds, Astbury Centre for Structural Molecular Biology, Leeds LS2 9JT, W Yorkshire, UK; 3 DNA Analysis and Protein Production Facility, Faculty of Biological Sciences, University of Leeds, Leeds, LS2 9JT, UK; 4 Institute of Molecular and Cellular Biology, Faculty of Biological Sciences, University of Leeds, Leeds, LS2 9JT, UK; 5 Astbury Centre for Structural Molecular Biology, University of Leeds, Leeds LS2 9JT, UK

## Abstract

**Background:**

Facilities that provide a service for DNA sequencing typically support large numbers of users and experiment types. The cost of services is often reduced by the use of liquid handling robots but the efficiency of such facilities is hampered because the software for such robots does not usually integrate well with the systems that run the sequencing machines. Accordingly, there is a need for software systems capable of integrating different robotic systems and managing sample information for DNA sequencing services. In this paper, we describe an extension to the Protein Information Management System (PIMS) that is designed for DNA sequencing facilities. The new version of PIMS has a user-friendly web interface and integrates all aspects of the sequencing process, including sample submission, handling and tracking, together with capture and management of the data.

**Results:**

The PIMS sequencing extension has been in production since July 2009 at the University of Leeds DNA Sequencing Facility. It has completely replaced manual data handling and simplified the tasks of data management and user communication. Samples from 45 groups have been processed with an average throughput of 10000 samples per month. The current version of the PIMS sequencing extension works with Applied Biosystems 3130XL 96-well plate sequencer and MWG 4204 or Aviso Theonyx liquid handling robots, but is readily adaptable for use with other combinations of robots.

**Conclusions:**

PIMS has been extended to provide a user-friendly and integrated data management solution for DNA sequencing facilities that is accessed through a normal web browser and allows simultaneous access by multiple users as well as facility managers. The system integrates sequencing and liquid handling robots, manages the data flow, and provides remote access to the sequencing results. The software is freely available, for academic users, from http://www.pims-lims.org/.

## Background

Routine, small scale sequencing projects are often supported by shared facilities that support Sanger sequencing technology. Normally, users submit their samples to the facility and get their results back *via *a local file server or DVD. A number of different computer systems have been developed to help sequencing facilities with the management and control of their workflows. These include Kaleidaseq [1], VSQual [2] or MAGIC-SPP [3]. Post-processing programs are also available for high throughput data, generated by "Next Generation" (NGS) sequencing techniques. Software includes preAssemble [4], Garsa [5], or Gene Projects [6] and can assist from assembly to annotation. There are also a number of tools for work with ESTs [7-9]. All have in common the quality trimming step, vector masking and the assembly of the EST data, but differ in implementation, data presentation, accessibility and storage. Although effective, many of the tools available are not designed for simultaneous use by multiple users [2,3]. At the same time, there are few systems to help with initial data collection from the users. Typically, spreadsheets are used to communicate information to and from a facility, but they are completed manually and therefore severely limit the throughput of a facility. To be cost efficient, sequencing facilities normally need to deal with DNA samples prepared by a variety of different protocols and are required to conduct a diversity of sequencing operations. Accordingly, automation has become a cornerstone in the successful delivery of results at reasonable cost. The widespread use of liquid handling robots in the sequencing laboratories has made the automation of sample preparation for the sequencing machines possible. However, to realise the benefits of such robotic combination fully, there is a need for data management system that organises the sample information together with the preparation of the data for use by the robots.

For example, the most cost-efficient approach often involves the use of multiple robotic systems from different manufacturers. Although desirable, integration of robots is often difficult due to software incompatibilities. While software is typically supplied with robots, they are at best only capable of managing their own data, which often contain details of little relevance to the end user. Moreover, data access is rarely controlled - an important feature for a multi-user facility - and data are rarely accessible remotely. An additional problem associated with the use of robots is that the data generated are often in a proprietary format, such that for a user to read them an installation of licensed software on the relevant client computer is required. In short, these systems are not designed to interact with the end user. There is therefore a clear need for a software system capable not only of integrating multiple robotic systems but also of managing sample information through a centralized, searchable, user-friendly data store.

In recent years, the biological sciences research community has developed new ways of addressing the challenge of handling of heterogeneous data in an organised manner. In particular, Laboratory Information Management Systems (LIMS) are increasingly employed to help with some of these tasks [2,10]. For example, dedicated LIMS are widely used to manage raw and processed data associated with DNA sequencing ventures such as genotyping [11] and genome annotation [12]. Increasingly, there is a need for such data not only to be recorded and managed but also to be made available to third parties such as collaborators and funding agencies. For example, in the Biotechnology and Biological Sciences Research Council BBSRC-funded Membrane Protein Structure Initiative (MPSI), which involves eight universities collaborating on the project, data produced at one site, such as the University of Leeds, have to be accessible to any other member of the consortium and indeed to the BBSRC funding agency. To achieve this, the MPSI consortium has employed a LIMS known as the Protein Information Management System (PIMS) [13,14] to record experimental information on the high throughput production and characterisation of membrane proteins. The present study describes the extension of this system for DNA sequencing facilities. The resulting system is an integral part of PIMS, allows the integration of liquid handling and sequencing robots and is capable of handling information on all stages of sample processing through a simple web interface. Despite the large number of tools that have previously been developed, the PIMS system described here is unique in its ability to interoperate with different robotic systems, cater for the data management needs of sequencing facility and provide a simple workflow from the initial data entry to the results collection.

## Implementation

The system was designed with two different sets of users in mind - *facility users *and *operators*. Facility users are the customers of the system and can easily navigate their samples or sequencing orders, regardless of when they were placed. Operators are the managers of the PIMS system and can store and access all the sample processing details from all facility users. Customers are thus shielded from an unnecessary amount of detail while the operators benefit from being able to capture all the sample processing details. To satisfy these differing needs optimally, the system displays different views of the same data to each type of user: a sample submission interface (customer and operator accessible), a sample processing interface (operator accessible) and a result retrieval interface (which is different for customer and operator).

The extension for the sequencing facilities is an integral part of PIMS, both systems share the same architecture and data model. The PIMS utilises a client-server architecture with standard internet browsers playing the role of the clients. It can be operated through any web browser and does not require any client programs to be installed on the users' computers. The sequencing extension front end is enhanced with the latest technologies such as AJAX for improved interactivity.

PIMS exploits J2EE technologies and the PostgreSQL database and is therefore very scalable and ready to store information on hundreds on thousands of samples. As PIMS was designed to work with many users, it has extensive user access configuration options. In the University of Leeds installation, we have a separate group for each laboratory which is using the system. Depending on the level of collaboration between the laboratories they may choose to be able to view/modify/delete each others' data or not. If they choose to have no access to the laboratory data of other groups, then the system ensures that they will never see these data.

## Results

### Sample submission

**Figure 1:**
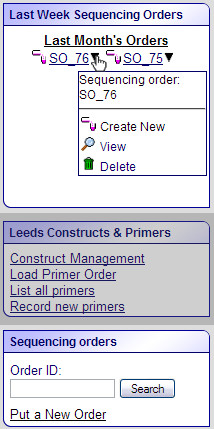
**A part of the screenshot of the main PIMS page**. The actions irrelevant to the functionality described are greyed out. The "Last Week Sequencing Orders" widget displays the list of orders submitted during last seven days by the current user. "Last Month's Orders" is an active link which takes the user to a page displaying the list of their orders submitted during the last month. The black triangle next to each sequencing order name gives access to a number of actions: creating a new order, viewing or deleting the order. The sequencing orders widget at the bottom of the screenshot allows a user to create and search the orders.

Upon logging into the PIMS, users can immediately create a new sequencing order and see their last week's orders in the right top corner of the main PIMS page (Figure 1).

One of the frequent irritations of large computer systems is the difficulty of locating the desired information. Accordingly, the PIMS sequencing extension is designed to display only the information relevant to the user. For example, when the user logs into PIMS, the main page displays the orders recently submitted by this user. Older orders or orders which do not belong to the user are not displayed. Normally, this filtering is sufficient to allow the user to find the information they are interested in. Alternatively, the results can be located by searching with the unique sequence order number. It is also possible to search for individual samples by any of their details, e.g. template or primer names, principal investigator or user names and so on. All the information in PIMS is seamlessly linked and the list of results is paginated to ease navigation.

**Figure 2:**
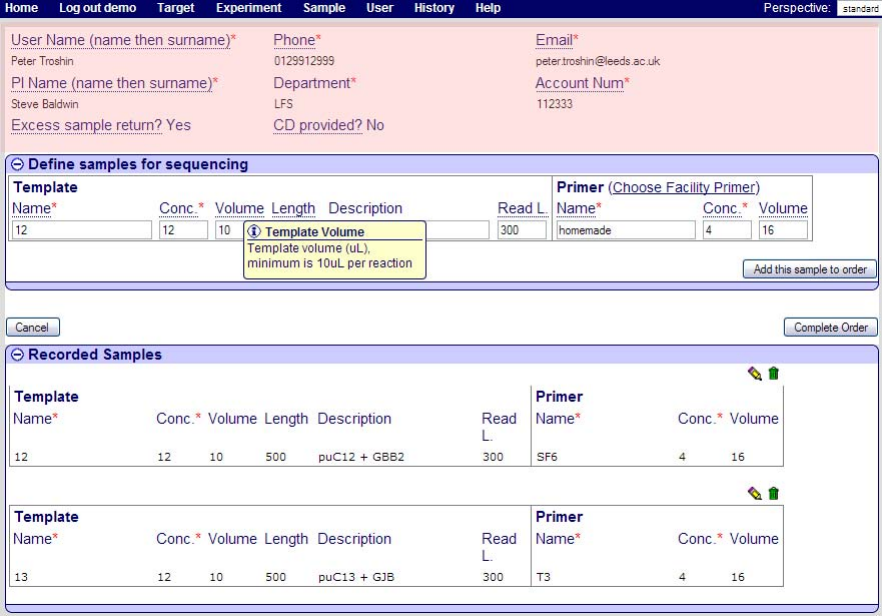
**Screenshot of the "create a new sequencing order" page in PIMS**. The section highlighted in pink defines the user contact and payment details, while the "Define samples for sequencing" area is for adding a new sample details. The "Recorded Samples" section lists samples already in the order. An extensive help text is displayed for each word underlined with a dotted line. Samples, which are already added into the order can be deleted by clicking on the green bin icon or amended by clicking on the pencil icon. A red star symbol next to field names denotes a required field.

Creating a new sequencing order in PIMS is straightforward; just fill in the web form (Figure 2).

The required information can either be input via a web submission form (Figure 2) or by uploading a spreadsheet. The spreadsheet method allows the rapid submission of up to 96 samples at once, and is aimed for frequent facility users who know the rules, such as required concentrations and minimum sample volumes. Many fields come pre-loaded with the most frequently used values or allow selection of pre-defined values from drop-down lists. The PIMS sequencing extension validates the data, as it is important that the concentrations and volumes of templates and primers lie within predefined limits.

Once the requisite sample information has been submitted, the user is given a unique order identifier similar to the order number familiar to users from online shopping. Using the order number, users can always retrieve information on the samples in the order. The order can be amended after submission, for example, by removing, adding or editing samples, so long as a sequencing operator has not started processing it. Along with the order id, the users are given a list of the templates and primers, plus details of the volumes that should be provided to the sequencing facility. The list of primer (if any) and template names with minimal volume to be provided to the facility is submitted to the user email address together with the order confirmation. This email contains interactive links to the appropriate pages within the PIMS system allowing the users to review their order. The last stage of the sample submission process is the submission of the physical samples for sequencing. The samples must be labelled according to the names provided to the PIMS and the order number must be provided. Optionally the order confirmation email can be printed and supplied with the samples.

### Sample processing

**Figure 3:**
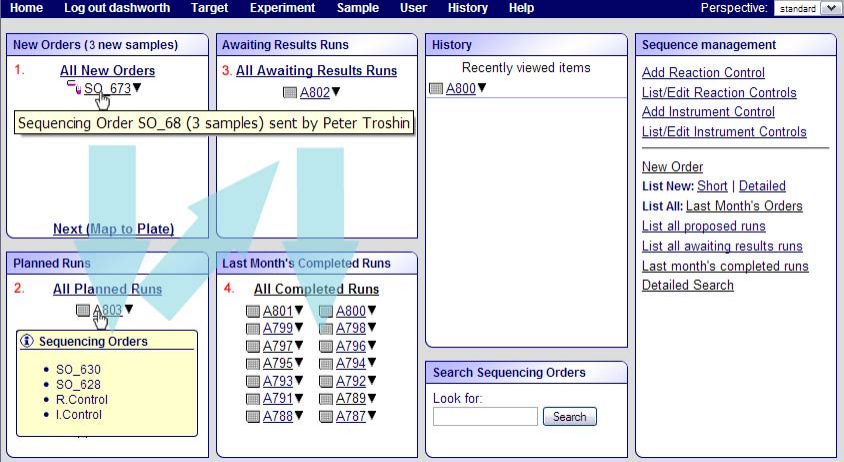
**Superposition of screenshots of the main page of the sequencing administrator interface**. The information in yellow tooltips is displayed only when the user mouses over the order (top page) or the run (bottom page). In the case of the order, the number of samples in the order and the name of the person who submitted the order is displayed in the tooltip. In the case of the run, the list of the orders that the run contains is displayed as well as the reaction (R.Control) and instrument (I.Control) controls if there are any. Green arrows emphasise the workflow, the orders from the top left (1), are grouped into the planned runs (2), then, the planned runs, progress to awaiting results runs (3), and finally upon uploading the results, the runs become complete (4). Numbers 1 to 4 as well as the blue arrows have been added manually to the screenshot for the illustration. The box entitled "History" displays the links to recent actions. The box "Search sequencing orders" is for searching the orders that are too old to be displayed in the completed run box. Finally, the sequence management box contains a collection of most frequently used action links.

Since the sample submission forms are completed before the sample arrives at the facility, the facility operator knows in advance what to expect and so can plan accordingly. Sequencing operators have additional facilities in PIMS to help them with the sequencing process. The main page for the facility operator contains an overview of all plates in all processing stages. It immediately shows how many runs are being processed, how many samples remain to be processed, as well as how long they have been in the queue (Figure 3).

For the sequencing facility operator, the sample processing is a four stage process. Each processing stage represents a distinct operation in PIMS and can be performed at a time convenient for the operator. The four stages are:

1) New Order

2) Planned Run

3) Awaiting Results Run

4) Completed Run

where a "Run" represents a plate, or part of a plate containing different samples, possibly from different users' or customers' orders. The main page for the sequencing administrator reflects these steps.

Facility users submit their samples to the facility and record their sample details into the PIMS system effectively creating the new orders. From the sequencing facility operator point of view the new order stage is there their work begins.

The "Planned Run" is a plan, mapping user samples to the sequencing plate wells. The "Awaiting Results Run" is a sequencing plate for which the full chemical composition of the wells is known, such that the plate can be prepared by the liquid handling robot and consequently processed by the sequencer. The "Completed Run" - is a run which has been processed, i.e. samples sequenced and the sequencing results are uploaded into PIMS. In other words the completed run is where the facility operator hands the work he or she has done to the customers. A detailed explanation of each of these stages is provided below.

Sample processing operations are available for the sequencing operators only. Ordinary users do not have access to this functionality and thus are shielded from unnecessary details. At the same time, facility operators are in a full control of the sample processing and can be certain that no one can alter or indeed even view the data. Data protection is an important issue for any facility operating on samples from multiple users.

### Planning the sequencing experiment

Planning of a sequencing experiment's functionality is only available to the sequencing operators. The sequencing operator plans the run from the sequencing administrator main page, which displays the list of orders to be processed, and calculates the total number of samples in single or multiple orders, as well as the number of plates required to sequence all the samples.

Samples need to be mixed with primers and other chemicals in a plate for sequencing. This can be done manually or using a liquid handling robot. The amount of sample in a well depends on its concentration and quality. To minimise the costs, the sequencing plate should have no or very few empty wells. At the same time, the users should not need to wait an excessively long time for their samples to be sequenced. Given the large number of samples processed by a typical sequencing facility, planning becomes a complicated process. In addition, one needs to keep track of all the samples on the sequencing plate to be able to match the sequencing results back to them. PIMS helps to meet these needs by proposing a plate plan automatically, taking into account all the variables mentioned above.

Once the planned run is recorded, any orders it contains can no longer be amended and are no longer displayed on the new orders lists. However, it is still possible to abandon the plan and start everything from scratch.

### Manual or Robotic handling of the samples

After the plate plan is completed and saved, it is displayed in the "Planned Runs" panel of the main sequencing administrator page. The generated map of the run contains the volumes of template, primer, reaction mix and water to be added to each well. This map can be generated either for manual processing in the form of an Excel spreadsheet or as a CSV file for robotic processing. The two templates differ mainly in the volumes used.

To avoid reprogramming robotic systems for every run, a time consuming task which is prone to errors, all the modern liquid handling systems have the ability to load CSV files. As stated previously, PIMS can output the list of volumes of primers, water, buffer and templates in this format. Each of the three different liquid handling robots used within the University of Leeds sequencing facility requires a different output format, reflecting the different layouts of the decks of the robots. The sequencing extension can automatically generate 3 different types of CSV files for different robots and sequencing types. The most frequently used CSV file has fixed volumes for templates and primers, which are the same for all wells. This allows for faster sample processing using a time-optimised program. However, in some cases it is important to be able to vary the volumes of primer and template for each well. PIMS can prepare a differently formatted CVS file for such cases. The operator can choose whichever option is the most appropriate for the samples.

As the qualities of the DNA or the primers provided by the customer can vary, the required volumes might need to be changed to optimise the sequencing reaction. To allow the sequencing operator to address this problem, the variable volumes CSV files as well as the spreadsheet for manual processing can be amended. The modified files can then be uploaded into PIMS to provide a record of the alterations.

The system also prepares the task file for the sequencer, designated the "instrument set-up sheet". This sheet contains concatenated sample and primer names for each well, a unique run number and a unique reference number for each sample. The sample reference number is searchable and represents a unique reference to a condition in a particular well. Once the sequencing set-up sheet is uploaded, the PIMS considers the run to be at the "Awaiting Results" stage.

### Completing the run

**Figure 4:**
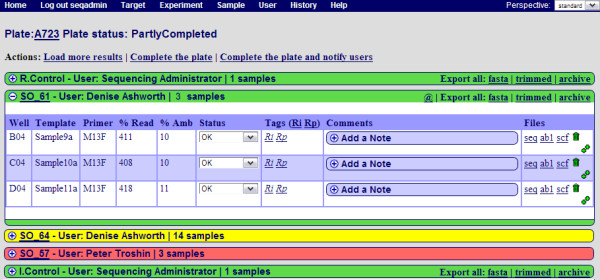
**Screenshot of the "review uploaded results" page**. This page lists the orders contained in the plate. All results, some results and no results, have been uploaded for the orders highlighted in green, yellow and red respectively. The order number, the name of the user who submitted the order and the number of samples in the order are displayed on the top information bar of each order. By clicking on the plus icon on the left side of each order the list of samples can be shown or hidden. When displayed, the position in the plate, the template and primer names, the percentage of the required read length (% Read), the percentage of ambiguous letters (% Amb), the status of the experiments (OK, Failed, In_Process), Tags (Rp - re-process and Rj - re-inject), notes and links to download individual sequencing files are displayed. "@" symbol denotes an action button notifying the user by email that results are ready for collection.

Once a prepared plate has been loaded into and processed by the sequencer, the sequencing results need to be uploaded into the PIMS system. The run is considered to be at "awaiting results" stage before this is done. The output of the sequencer consists of a set of files for each sample. It is possible to upload files for the same run one by one but for convenience the files are packed into a single ZIP archive which can then be uploaded into PIMS. The system then automatically links each result file from the archive with the sample information already recorded in the system. A "traffic lights" system of green, yellow and red colours respectively is used to indicate orders in which respectively all, some or no samples have been uploaded (Figure 4).

Upon uploading of the archive, the system rates the results either as OK, Failed or In_Process. OK means that the sequencing has been successful and the length of the sequence obtained is equal to or greater than that requested. Failed means that the sequencing has not been successful, or that the readable sequence length is shorter than that requested. In_Process marks the samples for which results have not been uploaded yet. The automatically assigned status can be manually altered by the facility operator. The result files marked Failed are not shown to the user, but can be preserved for future reference by the sequencing operator.

Apart from being able to download individual chromatogram and text files with the sequence for each sample (by clicking on seq, ab1 and scf file links), it is also possible to download all results at once. For example, option "fasta" exports all the sequences from the order as a FASTA formatted file. Option "trimmed", does the same, except it chops unresolved parts of the sequence from the ends, while option "archive", exports all the results that have been uploaded, including chromatogram files.

It is possible to annotate each sample with any number of notes, each of which bears the date and time when it was created and its author. These notes can either be visible to the sequencing operator only (private), or visible to everyone (public). Failed samples can either be tagged for re-injection or marked for re-processing if the cause of failure is unclear to the operator.

Once the sequencing operator has uploaded and scored all the results and added notes for the users, the plate can be completed. When the plate is completed the users are notified by email that their sequencing order is completed. The email contains a link to the web page where the results can be collected.

### Data retrieval

**Figure 5:**
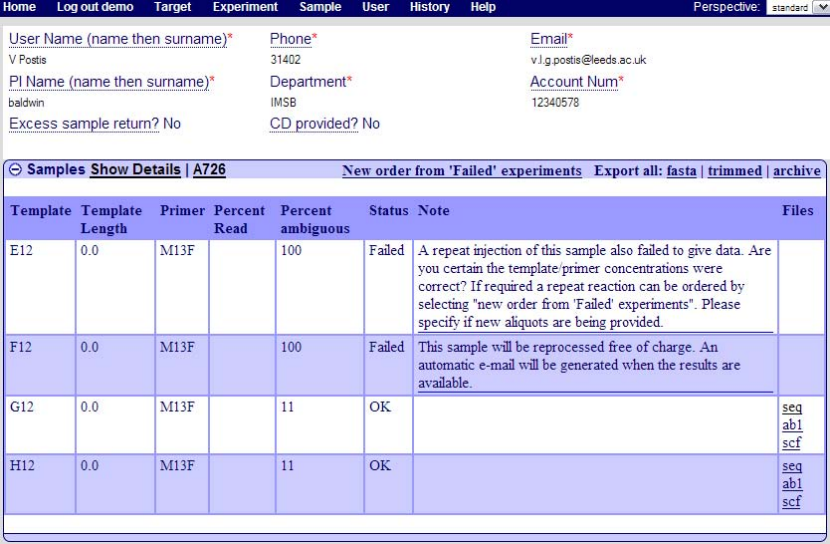
**Screenshot of the "Completed order" page as seen by the facility user**. Operators are presented with a more detailed view than a user. In particular, they can add/remove/change result files, statuses, tags and notes. The "Show Details" link takes the user to the original view of the order containing the information which they have submitted. The link next to it called "A726" is the name of the run this order is a part of. This link takes the operator to the plate where the order was processed.

Upon receipt of an email, informing the users of the completion of the sequencing order, they can retrieve their results from the system (Figure 5.). PIMS ensures data safety by restricting the users to their own results.

For each sample the user is provided with sequencing results statistics which allow a judgement on the quality of the sequencing to be made. Samples which sequencing failed can be submitted to re-processing at the click of a button, there is no need to manually re-enter the sample information into the system.

## Conclusions

Whatever technology is used, sequencing facilities need to handle many samples every day. Liquid handling robots have become indispensable for sample processing while the PIMS sequencing extension described here brings the benefits of heterogeneous data integration.

The advantages of the PIMS sequencing extension include:

1) simplifying communication between many operators involved in the various steps of a given sequencing task;

2) optimising workflow when samples come from various customers but are processed in the same plate;

3) keeping customers informed about the processing of their samples - sequencing results are stored online and are remotely accessible by the customers at any time;

4) ensuring that all information about the provenance and the processing of each sample has been retained and is easily accessible from any location at any time;

5) allowing rapid and accurate pinpointing of any problems, by providing a means of reviewing the whole process;

6) tracking of unsuccessful experiments can be used to improve methods;

7) helping to anticipate future demands and optimising processes by providing statistical analysis of the facility workload and workflows;

8) simplifying basic tasks such as location and tracking of samples.

In this study we have described the installation and use of the PIMS sequencing extension at the University of Leeds DNA Sequencing facility, where the system has been in operation since July 2009. It has completely replaced manual data handling and simplified the data management and user communication tasks. This facility is currently set up with 45 user groups. The users access the system by logging in with individual user names and passwords. Users belong to one or more groups and have access to the data from other users within the group they belong to. This allows confidentiality of group data to be maintained. Only the sequencing administrators have access to all the sequencing-related data within PIMS. Different views for users and administrators keep the display uncluttered and relevant for everyone.

Introduction of the PIMS sequencing extension into this moderately high throughput facility greatly improved its efficiency. The PIMS system can export/import information at every step of the process and although, initially designed for use with the specific machines available in the facility, is not closely tied to these and can be easily adapted for use with other sequencers or liquid handling robots.

Moreover, the users of the PIMS sequencing extension can benefit from all the other PIMS features, in particular its ability to manage all aspects of a protein production starting from cloning and DNA sequencing to protein expression, purification and functional characterisation [S. Baldwin, V. Postis, D. Ashworth, unpublished]. Finally, by providing an easy way of storing and accessing information, PIMS helps to meet the requirements of funding agencies for transparency and accessibility to data generated from publicly-funded projects [15].

## Availability and requirements

The application is freely available for academic users and can be downloaded from http://www.pims-lims.org website. A demonstration version of the application is available at http://www.mpsi.ac.uk:8080/demo. For the latter, the user login and password are both "demo", while the sequencing administrator login and password are "seqadmin". The main sequencing administrator page can be found at http://www.mpsi.ac.uk:8080/demo/read/AdminHome. Please take care to login as sequencing administrator before accessing this page.

**Project name**: PIMS

**Project home page**: http://www.pims-lims.org

**Operating system(s)**: Platform independent

**Programming language**: Java

**Other requirements**: Web Application Server compatible with Servlet 2.4 spec or higher. Postgres 8.1 or higher or Oracle 10 and higher, Java 1.5 or higher

**License**: CCP4 [16]

**Any restrictions to use by non-academics**: licence needed

## Competing interests

The authors declare that they have no competing interests.

## Authors' contributions

PVT designed and developed the system, based on the requirements expressed by DA, and VLGP. DA was responsible for the detailed requirements, VLGP made a major contribution in defining the robot communication requirements. SAB MGM and GJB contributed to the development of the manuscript. All authors read and approved the final manuscript.
